# [^18^F]AV‐1451 PET in behavioral variant frontotemporal dementia due to MAPT mutation

**DOI:** 10.1002/acn3.366

**Published:** 2016-10-18

**Authors:** W. Richard Bevan Jones, Thomas E. Cope, Luca Passamonti, Tim D. Fryer, Young T. Hong, Franklin Aigbirhio, Jillian J. Kril, Shelley L. Forrest, Kieren Allinson, Jonathan P. Coles, P. Simon Jones, Maria G. Spillantini, John R. Hodges, John T. O'Brien, James B. Rowe

**Affiliations:** ^1^ Department of PsychiatryUniversity of CambridgeCambridgeUK; ^2^ Department of Clinical NeurosciencesUniversity of CambridgeCambridgeUK; ^3^ Wolfson Brain Imaging CentreUniversity of CambridgeCambridgeUK; ^4^ Discipline of PathologyUniversity of SydneySydneyAustralia; ^5^ Division of Anaesthesia, University of CambridgeCambridgeUK; ^6^ Department of NeuropathologyAddenbrooke's HospitalCambridgeUK; ^7^ University of New South WalesSydneyAustralia; ^8^ Medical Research Council Cognition and Brain Sciences UnitCambridgeUK

## Abstract

The validation of tau radioligands could improve the diagnosis of frontotemporal lobar degeneration and the assessment of disease‐modifying therapies. Here, we demonstrate that binding of the tau radioligand [^18^F]AV‐1451 was significantly abnormal in both magnitude and distribution in a patient with familial frontotemporal dementia due to a MAPT 10 + 16C>T gene mutation, recapitulating the pattern of neuropathology seen in her father. Given the genetic diagnosis and the non‐Alzheimer's pathology, these findings suggest that [^18^F]AV‐1451 might be a useful biomarker in primary tauopathies. Largerscale in vivo and *post‐mortem* studies will be needed to assess the technique's specificity.

## Introduction

The pathogenic role of tau is well established in many neurodegenerative diseases. Until recently it has only been feasible to examine the morphology, intensity, and distribution of tau pathology *post‐mortem*. Several radiolabeled compounds have been developed with evidence of binding to intracellular aggregates of tau,^1^, ^2^ allowing potential visualization and quantification of tau pathology using positron emission tomography (PET).

There is strong evidence *in vivo* and *post‐mortem* that [^18^F]AV‐1451 binds paired helical filaments of tau in Alzheimer's disease (AD).^3^, ^4^ The distribution and magnitude of *in vivo* tau binding correlates with AD staging,^5^ and recapitulates the anatomical distribution of focal onset forms including logopenic aphasia 6 and posterior cortical atrophy.^7^ Binding to tau in primary, non‐AD tauopathies is less well established, with inconsistency between *in vivo* PET findings and *post‐mortem* analysis in progressive supranuclear palsy.^3^, ^8^, ^9^

Genetically determined tauopathies provide an important opportunity for validation of tau tracers. It has recently been demonstrated that in advanced dementia due to *MAPT* mutation, the regional [^18^F]AV‐1451 binding *in vivo* correlates strongly with the density of tau pathology *post‐mortem*, and with glucose hypometabolism*,* 10 although differentiation between patients was not established, in either distribution or magnitude of [^18^F]AV‐1451 PET. Here, we compare the magnitude and distribution of [^18^F]AV‐1451 binding in healthy controls to a patient with behavioral variant frontotemporal dementia (bvFTD) resulting from a 10 + 16C>T mutation in the microtubule‐associated protein tau gene (*MAPT*).

## Methods

### Family history

#### Proband

The patient presented aged 51 with 3 years of gradual change in behavior and difficulty managing daily affairs, apathy, reduced empathy, obsessional behaviors, rigid routines, hyperphagia, and weight gain. Examination revealed adynamic, empty speech with preserved grammar, reduced verbal fluency, anomia, semantic deficits, and surface dyslexia. Eye movements were normal. Praxis, cortical sensation, and visuospatial function were intact. There were no cerebellar or extrapyramidal features and no signs of anterior horn cell disease. She scored 36/100 on the Addenbrooke's Cognitive Examination (revised), and 3/26 on the frontotemporal dementia functional rating scale,^11^ indicating severe deficits. Magnetic resonance imaging revealed asymmetric, predominantly left‐sided, frontotemporal atrophy. She and her father had a 10 + 16C>T mutation of MAPT, with an H1H1 haplotype.

#### Father

The patient's father presented at age of 59 years with 10 years of insidious personality change and inappropriate behavior without insight. He was disinhibited, restless, hyperphagic for sweet foods, with cognitive rigidity, stereotyped behaviors, and later apathy. He had semantic memory impairment, poor verbal fluency, anomia, and surface dyslexia. Visuospatial function and orientation were intact. There were no ocular or motor abnormalities. His initial Addenbrooke's Cognitive Examination score was 77/100. MRI showed frontal and anterior temporal lobe atrophy, more marked on the left. He died aged 63.

**Figure 1:**
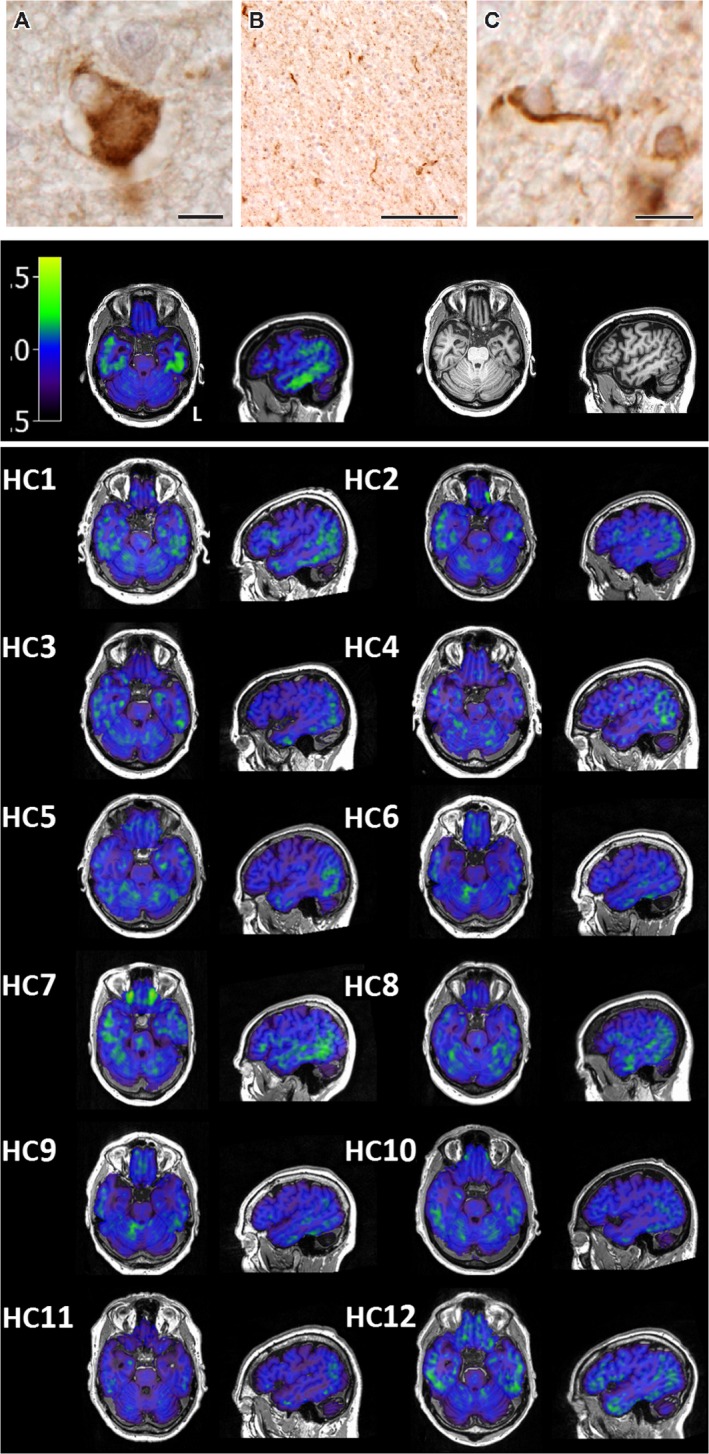
Upper panel: Representative neuropathological features of the proband's father's neuropathology in the superior frontal cortex showing a ballooned neuron (A), white matter threads (B), and a coiled body (C) immunostained with phosphorylated tau. Scale bar represents 20 *μ*m in (A), 100 *μ*m in (B), and 10 *μ*m in (C). Second panel: [^18^F]AV‐1451 BP_ND_ (left) and T1‐weighted MRI scan (right) for the proband. Lower panel: [^18^F]AV‐1451 BP_ND_ for each individual healthy control (HC). [^18^F]AV‐1451 BP_ND_ slices for all individuals are in the same position in native space.

Neuropathological examination (see Appendix for detail) showed moderate cerebral atrophy, most prominent in the frontal and temporal lobes, especially on the left. There was mild neuronal loss and gliosis throughout cortex, without neuritic plaques. Ballooned neurons were observed, immunopositive for phosphorylated 4‐repeat tau (Fig. 1A), as well as widespread thread pathology in grey and white matter (Fig. 1B), and coiled bodies in temporal lobes (Fig. 1C). Their morphology and distribution appeared typical for sporadic frontotemporal lobar degeneration with Corticobasal degeneration (CBD) pathology,^12^, ^13^ in keeping with MAPT mutation.^14^

#### Paternal grandmother

The patient's paternal grandmother developed a change in personality and behavior, with disinhibition, hoarding, and theft. Her death aged 51 was attributed to “cerebral atrophy”, without neuropsychological or *post‐mortem* examination.

### Positron emission tomography using [^18^F]AV‐1451

PET scanning used [^18^F]AV‐1451 and dynamic scanning over 90 min with a GE Advance scanner. A ^68^Ge/^68^Ga transmission scan enabled attenuation correction. Binding potentials, relative to a nondisplaceable compartment (BP_ND_), were determined from kinetic analysis with a simplified reference tissue model, using superior cerebellar grey matter as the reference region. In older subjects with variable perfusion, kinetic modeling overcomes the potential problem with standardized uptake value ratios arising from a failure to reach steady state. Brain parcellation used the Hammers brain atlas,^15^ expanded to include subcortical structures.^16^

### Data modeling and statistical method

Two questions were posed. Firstly, were there areas of the brain with higher BP_ND_ in the proband than 12 healthy adults (55–80, mean age 66, 50% male)? For each region, a robust t‐score was calculated for the patient compared to the control group, adjusting for the relatively small size of the sample.^17^ This converges with a similar Bayesian approach.^18^

**Figure 2:**
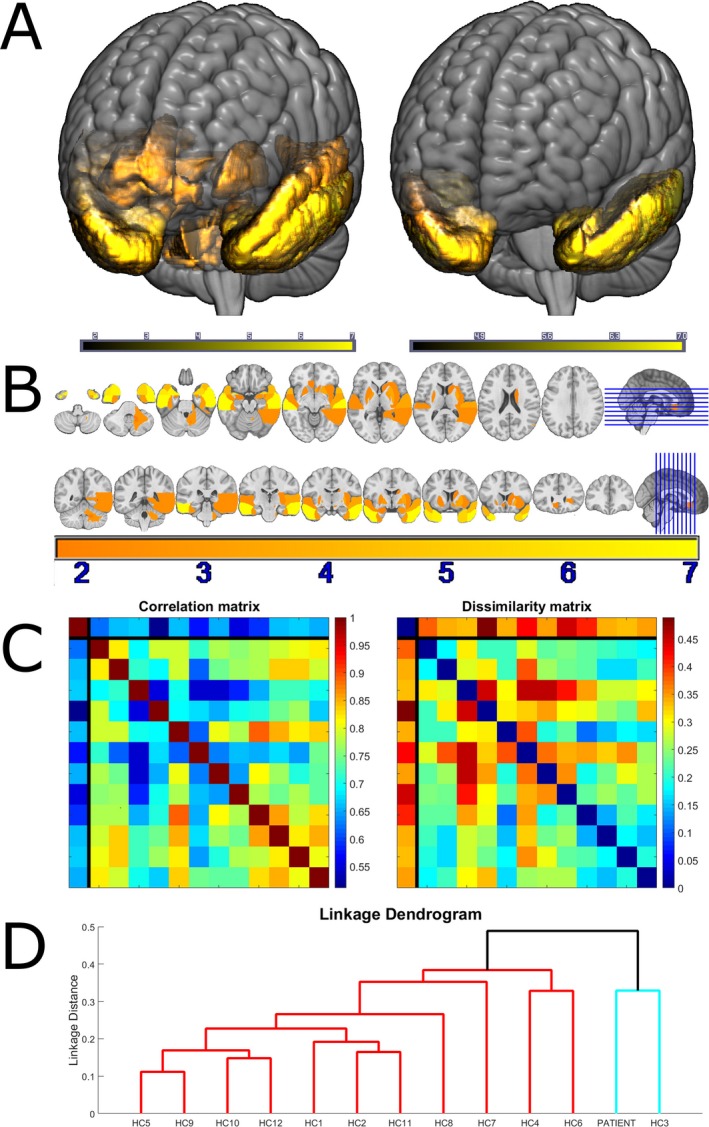
Panel A: A volumetric rendering of the MNI152 template MRI brain scan, overlaid with the t‐scores for ligand binding in those brain regions meeting a one‐tailed statistical threshold of *P* < 0.05, before (left) and after (right) correction for multiple comparisons. Panel B: coronal and axial sections through the template brain, overlaid with the same ligand binding t‐scores. Panel C left: Spearman correlation matrix between all individuals. The first row and column, separated by black lines from the other rows and columns, represents the patient. The other 12 columns represent controls. Panel C right: The same data expressed as dissimilarities (1 correlation). Panel D: the dendrogram produced by hierarchical cluster analysis. The two resultant clusters are colored in red and cyan. Controls are numbered according to their order in the upper panel correlation and dissimilarity matrices.

Secondly, irrespective of the absolute level of ligand binding, did the distribution of binding across brain regions differ between the proband and healthy adults? An hierarchical cluster analysis approach was used to answer this question. The parcellated [^18^F]AV‐1451 BP_ND_ data were converted into individual linear vectors by region of interest. These vectors were nonparametrically correlated (Spearman's rho), giving a correlation matrix (Fig. 2C), and converted into a dissimilarity matrix (1 correlation, Fig. 2C). Dissimilarity fed into hierarchical cluster analysis with thresholding for two groups using a complete (‘farthest neighbour’) method^19^; the most stringent linkage method for a single case (Fig. 2D).

## Results

Figure 1 contains raw maps of BP_ND_ for all individuals. Figures 2A and B illustrate the areas with significantly elevated t‐scores in the proband. Bonferroni correction for 83 regions‐of‐interest comparisons confirmed significant differences in inferior temporal lobe, and inferior and medial temporal pole bilaterally, as well as right superior temporal pole (Table 1). As an indication of the sensitivity of the ligand in these regions, Table 1 also includes a column of the maximum BP_ND_ in each region for any of the controls, as well as the mean and standard deviation across all controls. For the left inferior temporal lobe, the region with the highest t‐score, the control mean BP_ND_ was 0.0086 (standard deviation 0.0346), the maximum BP_ND_ observed in any of the controls was 0.0572, and the MAPT patient's BP_ND_ was 0.2928. The MAPT patient's BP_ND_ in this region was therefore 8.2 SD above the mean, and 5.8 times more unusual than any of the controls.

**Table 1:** Corrected t‐scores from brain regions with statistically significant ligand binding potential (BP_ND_) at *P* < 0.05 uncorrected

Hammers Atlas Brain Region	Simplified Name	Hammers Region Number	MAPT BP_ND_	Control BP_ND_ max	Control BP_ND_ mean	Control BP_ND_ SD	Corrected t‐score
‘G_tem_midin_l’	Left Inferior Temporal	**14**	**0.2928**	**0.0572**	**0.0086**	**0.0346**	**7.90**
‘Ant_TL_inf_lat_r’	Right Inferior Temporal Pole	**7**	**0.5925**	**0.1342**	**0.0557**	**0.0738**	**6.99**
‘G_tem_midin_r’	Right Inferior Temporal	**13**	**0.3367**	**0.0793**	**0.0140**	**0.0447**	**6.93**
‘Ant_TL_med_l’	Left Medial Temporal Pole	**6**	**0.3118**	**0.0690**	**0.0094**	**0.0441**	**6.59**
‘Ant_TL_inf_lat_l’	Left Inferior Temporal Pole	**8**	**0.3950**	**0.1245**	**0.0478**	**0.0540**	**6.18**
‘Ant_TL_med_r’	Right Medial Temporal Pole	**5**	**0.2774**	**0.0860**	**0.0071**	**0.0485**	**5.36**
‘G_sup_temp_ant_r’	Right Superior Temporal Pole	**83**	**0.4292**	**0.2080**	**0.1191**	**0.0705**	**4.23**
‘G_sup_temp_ant_l’	Left Superior Temporal Pole	82	0.3792	0.1995	0.0809	0.0721	3.97
‘Putamen_r’	Right Putamen	39	0.4353	0.3568	0.2369	0.0552	3.45
‘G_sup_temp_cent_r’	Right Superior Temporal	11	0.2122	0.1181	0.0377	0.0495	3.39
‘Subcall_area_r’	Right Ventral Anterior Cingulate Cortex	79	0.3919	0.2645	0.1296	0.0751	3.35
‘Pallidum_r’	Right Pallidum	43	0.5226	0.3514	0.2128	0.0995	2.99
‘G_occtem_la_r’	Right Perirhinal Cortex	15	0.2112	0.1277	0.0059	0.0660	2.99
‘Pallidum_l’	Left Pallidum	42	0.4468	0.3797	0.1909	0.0864	2.84
‘Cerebellum_wm_r’	Right Cerebellar Grey Matter	90	−0.0009	−0.0280	−0.0614	0.0219	2.65
‘G_occtem_la_l’	Left Perirhinal Cortex	16	0.1677	0.1223	0.0233	0.0531	2.61
‘Amygdala_r’	Right Amygdala	3	0.2248	0.1413	0.0535	0.0636	2.59
‘NuclAccumb_r’	Right Caudate	37	0.4768	0.4355	0.1970	0.1083	2.48
‘PosteriorTL_r’	Right Posterior Temporal	31	0.0934	0.0625	−0.0019	0.0386	2.37
‘G_sup_temp_cent_l’	Left Superior Temporal	12	0.1400	0.1135	0.0291	0.0452	2.36
‘Putamen_l’	Left Putamen	38	0.3947	0.3750	0.2463	0.0617	2.31
‘Insula_r’	Right Insula	21	0.1693	0.1198	0.0531	0.0493	2.26
‘S_nigra_r’	Right Midbrain	75	0.4506	0.3863	0.2332	0.0958	2.18
‘CaudateNucl_r’	Right Caudate	35	0.3383	0.3522	0.1738	0.0787	2.01
‘Subgen_antCing_l’	Left Ventral Anterior Cingulate Cortex	76	0.1090	0.0752	−0.0107	0.0578	1.99
‘Amygdala_l’	Left Amygdala	4	0.1800	0.1838	0.0514	0.0670	1.84
‘Cerebellum_dentate_r’	Right Cerebellar Dentate	239	0.0850	0.0697	0.0208	0.0342	1.80
‘FL_strai_G_r’	Right Medial Orbitofrontal Cortex	53	0.2492	0.2452	0.1297	0.0646	1.78

Regions surviving Bonferroni correction for 83 comparisons (*P* < 0.0006) are in **bold**. For each region, the BP_ND_ for the MAPT patient is given, along with the maximum BP_ND_ observed in any of the controls and the mean and standard deviation of BP_ND_ across all controls.

Hierarchical cluster analysis of the distribution (Fig. 2D) distinguished two groups. One (red in Fig. 2D) contained 11 of the 12 healthy elderly individuals, whereas the other (cyan in Fig. 2D) contained the patient and 1 of the healthy individuals. Cluster analysis, blinded by nonparametric methods to the degree of ligand binding, therefore, provided statistically significant classification (binomial *P* = 0.003). The control classified together with the patient under the farthest neighbor method was 80 years old. She did not display any cognitive abnormalities (ACE‐R 98/100, MMSE 30/30), but parametric t‐test comparison of this individual to the other controls revealed higher BP_ND_ in right hippocampus (t_10_ = 5.14; *P* < 0.05 Bonferroni corrected) and parahippocampal gyrus (t_10_ = 4.53; *P* < 0.05 corrected), but unlike the proband, the control's BP_ND_ was not elevated in inferior temporal lobes. Therefore, although the overall pattern of regional binding in this control was less dissimilar from the MAPT patient than it was from the most dissimilar of all of the other controls, there was a clear dissociation from the MAPT case. It is possible that [^18^F]AV‐1451 detected asymptomatic Alzheimer's disease pathology in this healthy control, as expected in a proportion of older adults. Validation linkage analysis with the ‘nearest neighbour’ method confirmed the patient distribution to be unique.

Together, these results indicate that it is not simply the case that the proband had globally elevated [^18^F]AV‐1451 BP_ND_, but that the BP_ND_ distribution was also significantly different reflecting frontotemporal lobar degeneration. The patient had particularly abnormal BP_ND_ in anterior temporal lobes and ventral anterior cingulate cortex; areas that are particularly prone to tau accumulation in frontotemporal dementia,^20^ and that were neuropathologically most abnormal in her father.

## Discussion

The main finding is of a dissociated increase in [^18^F]AV‐1451 binding potential in the anterior temporal lobes and ventral anterior cingulate cortex in a patient with *MAPT* mutation, recapitulating the distribution of neuropathology in her father. The mutation in this family leads to C>T change in the *MAPT* pre‐mRNA at position 16 of the splice donor site of intron 10. This results in the increased incorporation of exon 10 into *MAPT* mRNA, creating an accumulation of the 4‐repeat tau isoform neuropathologically resembling CBD.^21^

The validation of tau tracers would have major implications for diagnosis and clinical trials in disorders associated with frontotemporal lobar degeneration. PET studies in cases with *MAPT* mutations provide an important facet of such validation, especially in combination with a strong, clear history, and the availability of neuropathological confirmation.

The primary limitation of this study is that it does not address the specificity of binding of [^18^F]AV‐1451. This will require further work in other neurodegenerative disorders, notably those with TDP‐43 deposition.

In conclusion, the intensity and distribution of binding of the tau ligand [^18^F]AV‐1451 in a patient with a *MAPT* 10 + 16C>T mutation supports the use of this ligand in clinical studies of dementia, including frontotemporal lobar degeneration and CBS.

## Author Contributions

WRBJ and TEC jointly prepared the manuscript, undertook data analysis, and produced Figure 2, with the assistance of LP. PSJ produced the second and lower panels of Figure 1. TDF and YTH designed the acquisition protocol and preprocessed the data. JK, SF, KA, and MGS undertook the neuropathological and genetic examination, produced the upper panels of Figure 1, and authored the Appendix. FA, JC, JH, JOB, and JBR conceptualized and designed the study.

## Conflicts of Interest

JOB has acted as a consultant for GE Healthcare and Lilly.

## Macroscopic Observations

Following removal at autopsy, the brain was fixed in 15% neutral buffered formalin, and weighed 1420 g. The cerebellum and brainstem were separated from the cerebrum. Examination of the external surfaces found moderate cerebral atrophy, which was most prominent in the frontal and temporal lobes. The left temporal lobe was more severely affected than the right temporal lobe. The atrophy extended to involve the precentral gyrus and the superior parietal gyrus. The cerebral hemispheres were sectioned in the coronal plane. The cerebral atrophy observed externally was confirmed and there was enlargement of the lateral ventricles. The basal ganglia and thalami were unremarkable, although the subthalamic nucleus was slightly reduced in size. Horizontal sections of the brainstem revealed pallor of the substantia nigra and locus coeruleus. Apart from a small single infarct in the cerebellar cortex, the cerebellum was unremarkable.

## Tissue Preparation and Immunohistochemistry

Standard tissue blocks from the superior frontal, precentral, inferior temporal and anterior cingulate cortices, the hippocampus at the level of the lateral geniculate nucleus, the basal ganglia at the level of the head of the caudate nucleus, the midbrain at the level of the red nucleus, the pons at the level of the locus coeruleus, subthalamic nucleus, the medulla at the level of the hypoglossal nucleus, and the cerebellar dentate gyrus were embedded in paraffin wax and cut on a rotary microtome. Ten *μ*m sections were stained using hematoxylin and eosin and modified Bielschowsky silver. Immunoperoxidase staining using phosphorylated tau (clone AT8; mouse; 1:1000; Cat. No. MN1012; Thermo Scientific Australia, Scoresby, Victoria) was performed using a Discovery DX autostainer (Ventana Medical Systems, Tuscon, AZ, USA). 3‐repeat tau (mouse; 1:50; Cat. No. 05‐803; Abcam; Melbourne, Victoria), 4‐repeat tau (mouse; 1:50; Cat. No. 05‐804; Abcam), p62 (rabbit; 1:250; Cat. No. 610833; BD Biosciences; North Ryde, NSW), and alpha‐synuclein (mouse; 1:500; Cat. No. 610787; BD Biosciences; North Ryde, NSW) were performed manually. For antigen retrieval, 3‐repeat tau and p62 required heating in the pressure cooker at 110°C for half an hour in TE buffer (pH 9.0), 4‐repeat tau required pretreatment with formic acid for 15 min followed by heating in the pressure cooker at 110°C for half an hour in TE buffer, and alpha‐synuclein required pretreatment with citric acid buffer (pH 6.0) for 18 min in the microwave. Following blocking of endogenous peroxidase activity in 100% methanol with 3% hydrogen peroxide for 10 min, sections were blocked in 10% normal horse serum (NHS) in TBS buffer (pH 7.4). Primary antibodies diluted in TBS with 1% NHS were incubated at 37°C for 1 h, followed by incubation in EnVision Dual Link Polymer (Cat. No. K4061; DAKO; North Sydney, New South Wales). Dark brown staining was visualized by adding hydrogen peroxidase to a 3′3′‐diaminobenzidine solution. All sections were counterstained with hematoxylin.

## Microscopic Observations

### Histopathology

Sections of the cerebral cortex showed mild neuronal loss and gliosis in the superior frontal and inferior temporal cortices with the normal laminar distribution of neurons preserved. Ballooned neurons were observed in cortical sections stained with hematoxylin and eosin. Both the hippocampal CA1 region and entorhinal cortex showed mild neuronal loss and sparse neurofibrillary tangles. Neuritic plaques were not observed. Consistent with the macroscopic examination, there was mild neuronal loss of pigmented neurons in the substantia nigra and depigmentation in both the substantia nigra and locus coeruleus. The basal ganglia and cerebellum had minimal neuronal loss.

### Tau immunohistochemistry

Phosphorylated tau‐immunopositive ballooned neurons (Fig 1A), astrocytic plaques, widespread grey and white matter thread pathology (Fig 1B), and coiled bodies (Fig 1C) were observed in the superior frontal and inferior temporal cortices. The morphology and distribution of these inclusions appeared similar to sporadic frontotemporal lobar degeneration cases with CBD pathological subtype.^12^, ^13^ Immunostaining with 4‐repeat tau and p62 labeled a similar number of ballooned neurons, astrocytic plaques, and coiled bodies. Both 4‐repeat tau and p62 only labeled a small proportion of phosphorylated tau‐immunopositive threads in both grey and white matter. 3‐repeat tau immunoreactivity was not observed. Phosphorylated tau‐immunopositive Pick bodies and tufted astrocytes were not observed. Immunostaining with alpha‐synuclein was not observed in the cortex, hippocampus, or brainstem. The severity of neuropathological features immunostained with phosphorylated tau was similar in both the superior frontal and inferior temporal cortices with a mild number of ballooned neurons and astrocytic plaques, and moderate‐to‐severe coiled bodies and thread pathology in grey and white matter of both regions.
